# Infiltrating Ductal Breast Carcinoma in a Male Patient With Associated BARD1 Mutation

**DOI:** 10.7759/cureus.66216

**Published:** 2024-08-05

**Authors:** Joseph McGrath, Dillin J Rhatigan, Danielle Donahue, Mackenzie Fannin, Nuria Lawson

**Affiliations:** 1 Dr. Kiran C. Patel College of Osteopathic Medicine, Nova Southeastern University, Davie, USA; 2 General Surgery, Palmetto General Hospital, Miami, USA

**Keywords:** male breast cancer, bard1 mutation, bard1, general surgery, infiltrating ductal carcinoma (idc), modified radical mastectomy (mrm), alnd: - axillary lymph node dissection, rare gene mutation, mutyh mutation

## Abstract

Male breast cancer is an uncommon diagnosis with limited research on management and prognosis due to its rarity. We discuss a case of a 55-year-old male with a non-contributory past medical history who presented with an enlarging palpable mass of his right breast tissue at the 10:00 position. The ultrasound of the right breast showed a 2.8 cm heterogenous mass with irregular borders highly suspicious for malignancy. The follow-up sonogram-guided core biopsy was performed, and the pathology of the mass confirmed high-grade infiltrating ductal carcinoma. A modified radical mastectomy of the right breast with extensive axillary lymph node excision was performed. Genetic testing of the excised tumor revealed a MUTYH gene mutation and a BARD1 (BRCA1-associated RING domain 1) gene mutation of unknown significance. Histopathological analysis confirmed a Grade 2, ER/PR-positive, KI 67-positive, and HER2-negative tumor.

## Introduction

Male breast cancer is a rare diagnosis, comprising only 1% of all reported breast cancer cases in the United States [1]. The diagnostic and treatment protocols for male breast cancers are limited due to the rare occurrence of the disease and, therefore, a small sample size. Most treatment plans for male breast cancers, including the use of aromatic inhibitors and radical mastectomies, are extrapolated from the current literature on female breast cancers [2]. Risk factors for breast cancer development in males and females include older age, smoking, and alcohol consumption. The risk factors that are unique to males include cirrhosis, obesity, Klinefelter syndrome, and testicular injury [3].

The most well-studied mutations in breast cancer pathophysiology for both males and females are BRCA mutations [2]; however, a variety of mutations can lead to oncogenic changes in breast tissue. In addition to BRCA mutations, the most common genetic mutations that predispose males to breast cancer are CHECK2, MLH1, MSH2, and MSH6 [3]. We present a case of a 55-year-old male who was diagnosed with high-grade infiltrating ductal carcinoma of the breast following a sonogram-guided core biopsy of a right breast mass concerning for malignancy. The genetic testing of the tumor revealed a MUTYH gene mutation, which has been classically linked to colorectal and gastrointestinal cancer pathways [3], as well as a mutation in the BARD1 (BRCA1-associated RING domain 1) gene, which is classified as a mutation of unknown clinical significance currently.

Mutations in the MUTYH gene have been identified in patients diagnosed with familial adenomatous polyposis (FAP) but were not found to have the adenomatous polyposis coli (APC) gene mutation that is classically associated with the disease [4]. FAP with confirmed APC mutation predisposes patients to developing colorectal carcinoma. Furthermore, certain pathogenic variants of MUTYH, especially the p.Tyr179Cys variant, may contribute to an overall increased risk of developing breast cancer in males [odds ratio (OR): 4.54; 95% confidence interval (CI): 1.17 - 17.58; p = 0.028] [5]. However, the studies on this pathogenic variant require a larger sample size to confirm the potential association between MUTYH variants and male breast cancer.

The BARD1 gene has been identified as a tumor suppressor gene in the BRCA1-dependent and BRCA1-independent pathways [6]. Therefore, mutations in BARD1 have been postulated to confer oncogenic functions and predispose patients to developing breast cancer [6]. BARD1 mutations have been identified in prostate and ovarian cancer without significantly affecting the risk of developing either cancer [7,8]. However, the mutation is associated with an increased breast cancer risk in female relatives of the male carrier of the mutation [9]. The significance of a BARD1 gene mutation in male breast cancer management and prognosis is undetermined; however, the current literature suggests that the gene may play a vital role in male breast tumorigenesis by affecting the pathways previously mentioned.

This report, highlighting a case of male breast cancer, contributes to the ongoing discussion surrounding the role of BARD1 and its variants in breast cancer development. Enhanced understanding of such mutations would enable targeted diagnostic and treatment strategies while emphasizing the role of genetic testing in assessing potential cancer risk within families.

## Case presentation

**Figure 1:**
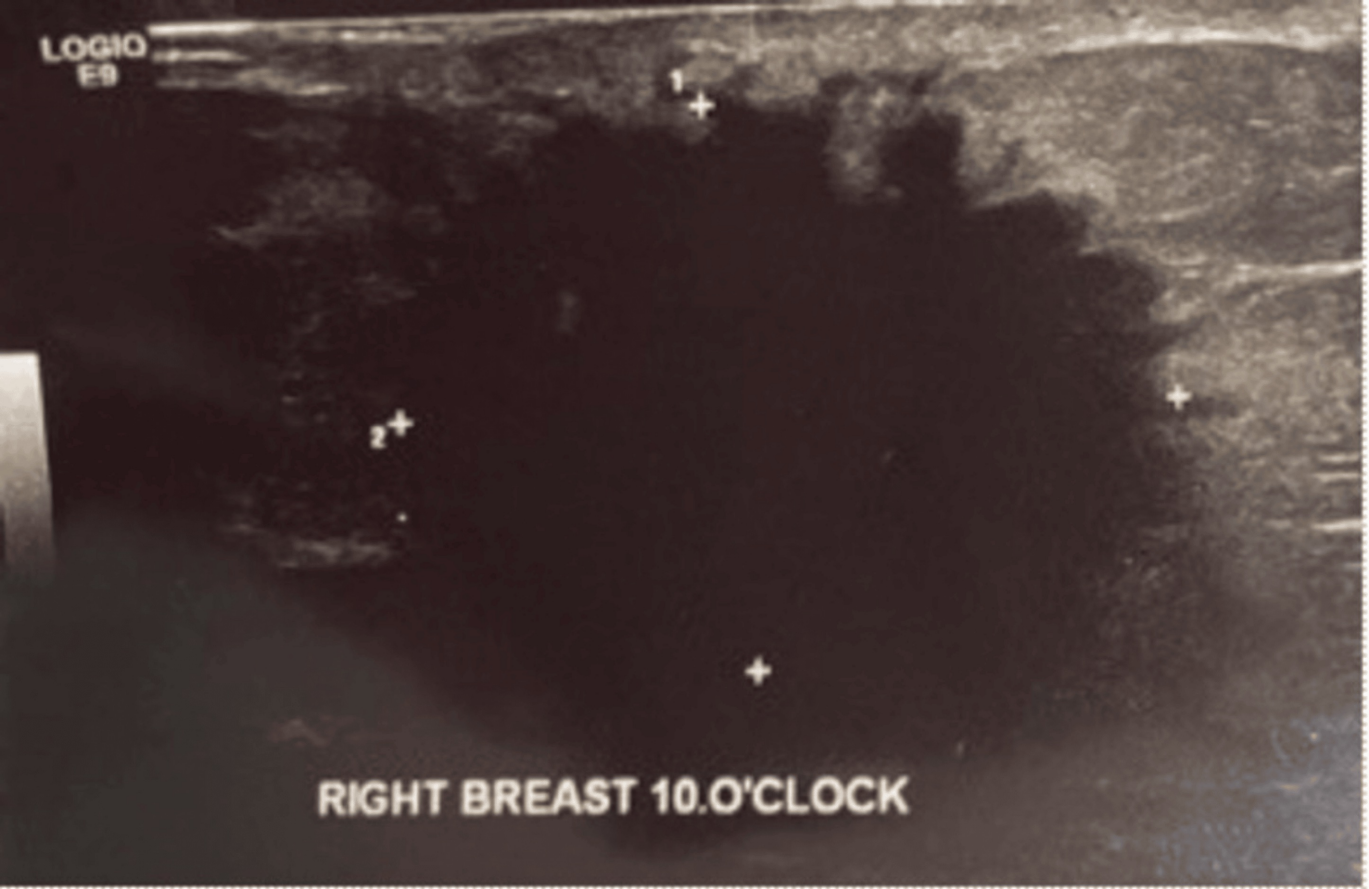
Ultrasonography of the 2.8 cm right breast heterogenous mass with irregular borders

A 55-year-old male with a non-contributing past medical history presented to the office with a palpable mass on his right breast that had been increasing in size over the past four months. The family history is significant for cervical and lung cancer in the patient’s mother, but the patient was unaware of the specific types of each cancer. He denied any family history of breast and ovarian cancer as well as a history of alcohol, tobacco, and illicit drug use. The patient had a BMI of 32.7. On the initial examination, a mass was palpated at 10:00 in the right breast that was indurated, mobile, and non-tender. No other masses were appreciated on the breasts, bilaterally. A sonogram in the office revealed a 2.8 cm irregular, highly suspicious mass at 10:00 in the right breast (Figure 1).

A sonogram-guided core biopsy of the suspicious mass at 10:00 in the right breast was performed and six core samples were taken. Pathology from the core needle biopsy revealed invasive ductal carcinoma with an overall grade of 2 of 3, involving all six core biopsies. The largest focus of invasive carcinoma measured on a single core involved by the tumor was 1.2 cm. A right radical mastectomy was recommended, and the patient consented. On the day of surgery, the patient was brought into the operating room after having been injected with four equal amounts of radioactive tracer into the right breast at the 3:00, 6:00, 9:00, and 12:00 positions in the nuclear medical suite for a total volume of 0.4 mL. The injection count of radioactive tracer in the right breast was determined to be 2,000 units via a Neoprobe.

**Figure 2:**
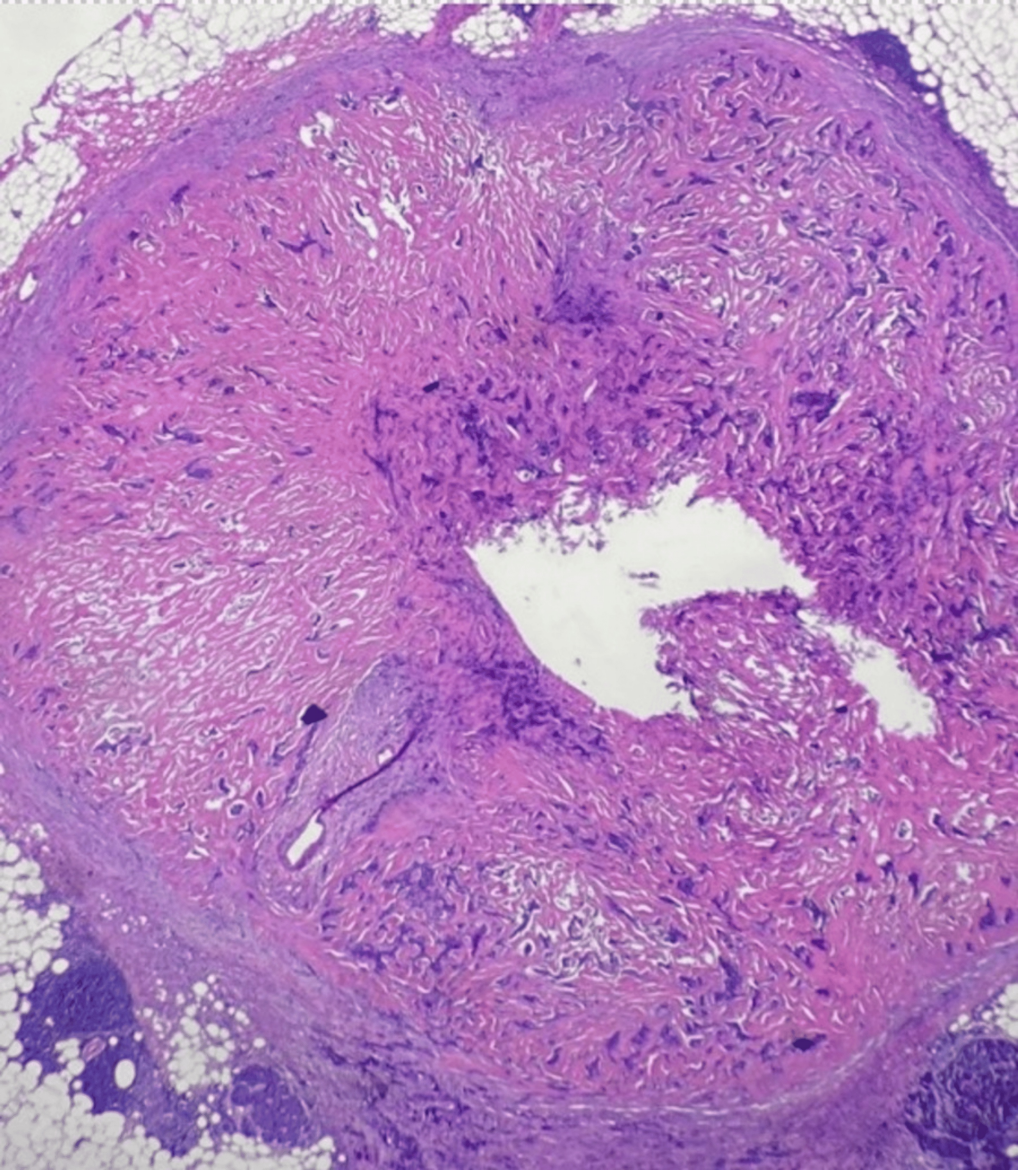
Right sentinel lymph node involvement by metastatic invasive ductal carcinoma

The transdermal counts were 200 in the right axillary region with a single localized focus compatible with radioactive tracer uptake within a sentinel lymph node. The transdermal count being at least 10% of the injection count indicated sufficient absorption of the radioactive tracer to be used for identifying possible malignant tissue intraoperatively. An incision was made elliptically from the parasternal area to the axilla using a skin knife encompassing the entire right breast. A Neoprobe was used as guidance to the area in the right axilla that had the maximum count. During the mastectomy, two hard indurated sentinel lymph nodes in the right axillary region adjacent to each other were identified, which measured 12 and 16 mm in their greatest dimension. Both lymph nodes were excised using the Bovie cautery. Neither of these lymph nodes had any counts but were concerning for metastasis and appeared to be replaced with cancer. The lack of radioactive tracer in the two sentinel lymph nodes was likely due to the tumor infiltrating the local lymphatic vessels blocking the flow of the radioactive tracer. The frozen section of these two lymph nodes revealed metastatic carcinoma to the axilla, prompting a complete axillary dissection (Figure 2).

**Figure 3:**
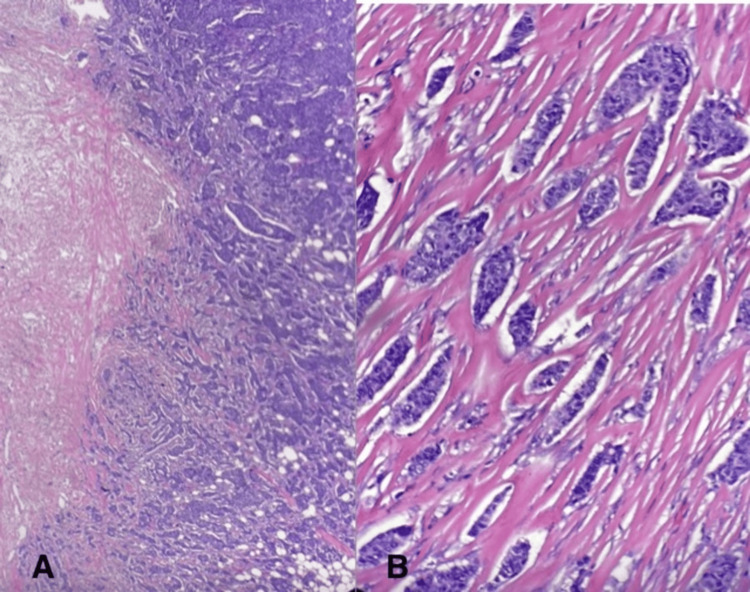
Histologic staining of right breast mass revealing high-grade invasive ductal carcinoma at 100x (A) and 400x magnification (B)

Tissue samples of lymph nodes and breast tissue collected during surgery revealed infiltrating ductal carcinoma overall grade 2 of 3, modified histologic grade of 6 (Nottingham Histologic Score): glandular differentiation score of 3, nuclear pleomorphism score of 2, and mitotic rate score of 1. The pathologic stage of the breast mass was pT2, pN2a (Figure 3).

Additionally, carcinoma was shown to directly invade the dermis or epidermis without skin ulceration. The breast tissue removed weighed 562 g and the breast tumor measured 4.7 cm in maximal gross dimension. Immunohistochemical stains of the breast mass determined the patient to be positive for ER, PR, and KI67, and negative for HER2. All margins were negative for invasive carcinoma; 21 lymph nodes were removed. Three of the removed lymph nodes were positive for macrometastatic carcinoma. Genetic testing revealed a heterozygous pathogenic variant, c.1187G>A (p.G396D), in the MUTYH gene seen in MUTYH-associated polyposis and colorectal cancer. Genetic testing also revealed a heterozygous variant of uncertain significance, c.1339C>G (p.L447V), in the BARD1 gene. The patient experienced no complications from the procedure and subsequently achieved a full recovery.

## Discussion

Breast cancer in men accounts for roughly 1% of all breast cancer diagnoses in the United States [1]. Additionally, the role of pathogenic variation of the BARD1 gene in the development and prognosis of breast and other cancers is an ongoing debate requiring further studies to determine the mutation’s clinical significance. Wild-type BARD1 is known to function as a tumor suppressor through pathways that are BRCA1-dependent and independent. The BARD1 protein forms a heterodimer with BRCA1 via N-terminal RING finger domains that, together, are vital in the DNA damage response pathways influencing the activity of ubiquitin ligase to encourage ubiquitination [6]. Through this interaction, the BARD1-BRCA1 complex has an essential role in preserving genomic stability within the cell [7]. This case report involves a 55-year-old male presenting with a right breast mass with a family history significant for cervical and lung cancer. The sonographic core needle biopsy revealed infiltrating ductal carcinoma leading to a prompt right radical mastectomy. During the mastectomy, two indurated axillary lymph nodes and breast tumor tissue were excised for pathological workup, revealing high-grade infiltrating ductal carcinoma. Genetic testing indicated mutations for MUTYH, which is associated with colorectal/gastrointestinal cancer, and a variant of unknown significance in BARD1. Due to the rarity of breast cancer in men and the lack of clinical studies on the pathogenic variants of BARD1, this case presents an opportunity to further examine the potential role of this gene in breast cancer diagnosis, management, and potential associations with other cancers. Much of the standard of care management of male breast cancer is extrapolated from clinical research in female breast malignancy patients for this reason. Recent studies on the mutation report low confidence in the risk of developing breast and other cancers as well as the prognosis due to the low sample size, especially of male breast cancer cases [7]. Also, there is minimal data on mutations of BARD1 and their clinical significance. Well-studied mutations, such as BRCA1 and BRCA2, have clinical evidence supporting platinum chemotherapies to provide effective management of the disease process. However, there are no similar studies on the optimal chemotherapy and post-surgical management for BARD1 cases [10]. Currently, surgical resection of the tumor is the standard of care and there is a lack of clinical studies supporting a particular chemotherapy regimen and/or post-surgical management specific for BARD1 mutation in breast cancer. The goals of this report are to emphasize the potential clinical significance of BARD1 mutation in breast cancer and to pursue a more tailored medical management to this mutation and genetic testing in family members to assess future risk of malignancy. The latter is important for female relatives due to recent research indicating an estimated two-fold increased risk for breast cancer and increased breast cancer risk among female relatives of a male carrier of the mutation [7,9].

## Conclusions

Breast cancer diagnosis in males remains extremely rare worldwide; however, the unique pathophysiology of rare genetic mutations found in male breast tumors requires further investigation. This report highlighted possible novel oncogenic mutations in the BARD1 gene, leading to breast cancer. Surgical resection of the tumor, breast tissue, and sentinel lymph nodes remains the current standard of care for male breast cancers, with the possibility of systemic chemotherapy or targeted hormone therapy dependent on multidisciplinary and patient-specific discussions. Further studies focusing on male breast cancer patients are required to improve prognosis and overall management.
